# Characterization of respiratory particles released during continuous speech and its relation to mask performance

**DOI:** 10.1038/s41598-025-97845-z

**Published:** 2025-04-16

**Authors:** Veronika Groma, Máté Vörös, János Osán, Balázs G. Madas, Árpád Farkas, Szilvia Kugler, Veronika Müller, Attila Nagy

**Affiliations:** 1 Institute for Energy Security and Environmental Safety, HUN-REN Centre for Energy Research, POB 49, Budapest, 1525 Hungary; 2 HUN-REN Wigner Research Centre for Physics, POB 49, Budapest, 1525 Hungary; 3 Department of Pulmonology, Semmelweis University, Budapest, 1085 Hungary

**Keywords:** Exhaled particles, Human lung, Facemask efficiency, Particle size distribution, Environmental impact, Health policy, Health care

## Abstract

Revealing the physicochemical characteristics of exhaled particles is essential for understanding and efficiently mitigating the airborne spread of contagious human illnesses. Among the most pivotal factors, the number size distribution of emitted particles plays a crucial role when considering atmospheric dispersion. This study focuses on submicron particles emitted during speaking, with particular attention on the changes over time. Moreover, the real-world (source control) efficiency of three types of commonly used facemasks (FFP2, surgical and 2-layer cotton mask) under in vivo conditions was studied. A specially designed cabin ensured a controlled environment, where a set of experiments was conducted on 28 participants. Our findings revealed no substantial variability in the number size distribution among different individuals and pitches. However, the quantity of emitted particles varied significantly among individuals, with differences reaching nearly two orders of magnitude. Additionally, the emitted number of particles strongly depended on the speaking volume, decreasing as speech volume was reduced. Submicron particles originating from the lungs and upper airways exhibited a consistent bimodal pattern, with peaks around 300 nm and below 100 nm. FFP2 and surgery masks worn by the subjects demonstrated robust performance in real-world conditions characterized by 80% source control efficiency even for the smallest particle size ranges tested. At the same time, textile masks yielded less favourable results of 50–60% source control efficiency.

## Introduction

The characterization of exhaled aerosols is of paramount importance to public health as pathogens can be transmitted via aerosols^1,2^. This entails that the understanding of their atmospheric dispersion is unavoidable. Advances in measurement technology have led to numerous studies during the last decades that have investigated the size distribution and other pertinent properties of aerosol particles, which have come even more into the focus of interest in the sight of the COVID-19 pandemic.

The key factors in assessing the risk of disease transmission via human exhaled particles are particle size and emission factor, that is, the mass of the pollutant divided by the duration of the activity performed by individuals suspected of being infected.

The generation of exhaled particles in humans is a complex process involving multiple mechanisms. The Bronchiole Fluid Film Burst (BFFB) mechanism produces small particles (< 1 μm) deep within the lungs as liquid films collapse during respiration^3^. As the bronchial tree, including small airways, distally narrows and partially closes during exhalation—especially in conditions like chronic obstructive pulmonary disease (COPD)—airway walls come into contact, obstructing airflow. During inhalation, the bronchioles re-expand, spreading a layer of epithelial lining fluid (ELF) that forms liquid menisci. As these layers rupture, tiny particles are generated, some of which are expelled during exhalation. This process actively contributes to particle production in various respiratory activities, including speaking and coughing^4^.

In addition to BFFB, the larynx plays a crucial role in particle generation through vocal fold adduction and vibration during actions like speaking, singing, coughing, and sneezing^5^. The narrowing of mucus-covered folds creates a flow restriction, leading to droplet detachment due to shear stress at the ELF-air interface. Dynamic ELF-coated fold movements further enhance particle formation through surface instabilities. Similarly, ELF films may burst, or ELF filaments may fragment as the glottic structure opens and closes^5^. Larger droplets primarily form in the upper respiratory tract—especially in the oral cavity and nasal passages—where saliva fragmentation, lip, tongue, and mouth movements contribute to droplet generation^6,7^.

Further insights into the internal sources of particles can be gleaned from Morawska et al.^4^, while Wang et al.^8^ provide a foundational perspective on airborne infection transmission via aerosols.

The refinement of exhaled particle characteristics occurred in parallel with the advancement of measurement technology. The initial in vivo measurements aiming to study the exhaled respiratory particles were primarily focused on the quantitative determination of particles exhaled as a result of human breathing activity and then extended to a wider and wider range of sizes and human samples, discussing the effect of environmental conditions^3,5,9–13^. Since the number of particles exhaled by humans is several orders of magnitude lower than the average concentration of environmental particles^11^, a dedicated measurement setup must be used for in vivo measurements that clearly distinguish emitted particles from background ambient particles. Various measurement setups have been used in the literature, either by creating a clean room environment^3,11,14^, by using a device intake point placed at the emission point^15–17^, collecting the particles to reach the detection limit of instrumentation^9^, or by combining these approaches^13^.

A recent review by Pöhlker et al.^18^ presented a comprehensive summary of the knowledge on the exhaled particles from the human lungs based on prior measurements. According to this summary study, it can be concluded that the size distribution of emitted particles exhibits a multimodal lognormal distribution during normal breathing. However, when the human emitter performs a speaking or singing activity, there is a significant increase in particle concentration in the size range below 10 μm as a combination of 3 modes (D_i_ = {0.13, 0.3, 1.1 μm}), the two bronchiole mode particles supplemented by trachea mode particles^18^. Additionally, based on the results of multimodal distribution functions fitted to size distributions of emitted particle number concentrations gathered from the literature^21^, it can be concluded that a 0.05 μm mode can also be distinguished. However, very limited measurement data is available for the size range below 300 nm.

Subsequent research focused on factors like individual variability, speaking/breathing modes, and noise level dependence due to instrumentation typically examining particles larger than 500 nm^7,14–17,19^. It became evident that human emissions exhibit substantial individual variability, with Asadi et al.^5^ identifying super-emitters and Mürbe et al.^13^ establishing a correlation between emitted particle quantity and speech noise level. Hartmann et al.^20^ demonstrated that more than 80% of the total number of bioaerosols released during breathing or various speaking modes fall within the size range below 1 μm.

Although numerous studies have delved into this area, yielding insights into particle size and concentration during various breathing activities and transmission mechanisms, critical details remain unresolved^21^. Different studies have different limitations, including focus on narrow particle size ranges, lack of direct concentration measurements, absence of controlled environmental conditions, limited size resolution data, and inadequate information for reliable particle size distribution determination. Additionally, studies often involve few subjects, omit investigation of vocalization-related activities, and lack data for children and adolescents. Bagheri et al.^21^ also presented data over the entire particle size range for 132 healthy volunteers aged 5 to 80 years and provided evidence that particles with different diameters originate from different parts of the respiratory tract. Particles with a diameter below 5 μm are generated in the lower respiratory tract, particles with a diameter between 5 and 15 μm in the larynx/pharynx, and particles larger than 15 μm are formed in the oral cavity. In addition, they provided information on the size distributions of exhaled fine and ultrafine particles.

Reducing the risk of human emissions is critical in terms of airborne infection spread, and one way to achieve this is through the use of masks. One of the focus points of the research on facemasks is the analysis of mask efficiency. The filtration efficiency of masks has been evaluated through various standards and studies^22–24^, mostly via laboratory experiments, rather than in vivo settings. Several methodologies assess mask efficiency between 300 nm and 10 μm, Joo et al.^25^ for example examined 33 mask types and identified low filtering efficiency in the 100–300 nm range.

Our research objectives encompass studying submicron (100–1000 nm) particle emissions during speaking based on in vivo experiments with a substantial sample size. A key hypothesis of our study is that speech-related respiratory particle emissions exhibit significant variability over time, influenced by factors such as speech volume and individual physiological differences. Given the limited number of previous studies with high-resolution temporal data, our findings provide valuable new insights into the temporal dynamics of bioaerosol emissions during continuous speech. In the case of respiratory particles, these findings constitute noteworthy supplements to the existing rare measurement results from the literature.

Furthermore, another central hypothesis of our research is that real-world wearing habits strongly influence the effectiveness of masks in source control for reducing emitted particles. By accounting for different ways individuals wear masks, we aim to provide a more realistic assessment of their protective efficiency, bridging the gap between idealized laboratory conditions and practical usage scenarios.

To test these hypotheses, we designed and built a dedicated measurement cabin that creates a high-purity environment by filtering surrounding particles, ensuring comfortable positioning for the participant, and allowing for the implementation of longer experimental series. Thanks to the special experimental setup and the opportunity of high time-resolution data detection, we discuss the time dependency of emitted bioaerosols during continuous speaking. A critical aspect of our study is the high time-resolution data acquisition, which facilitates the analysis of short-term fluctuations in bioaerosol generation.

## Methods

### Measurement setup

**Fig. 1:**
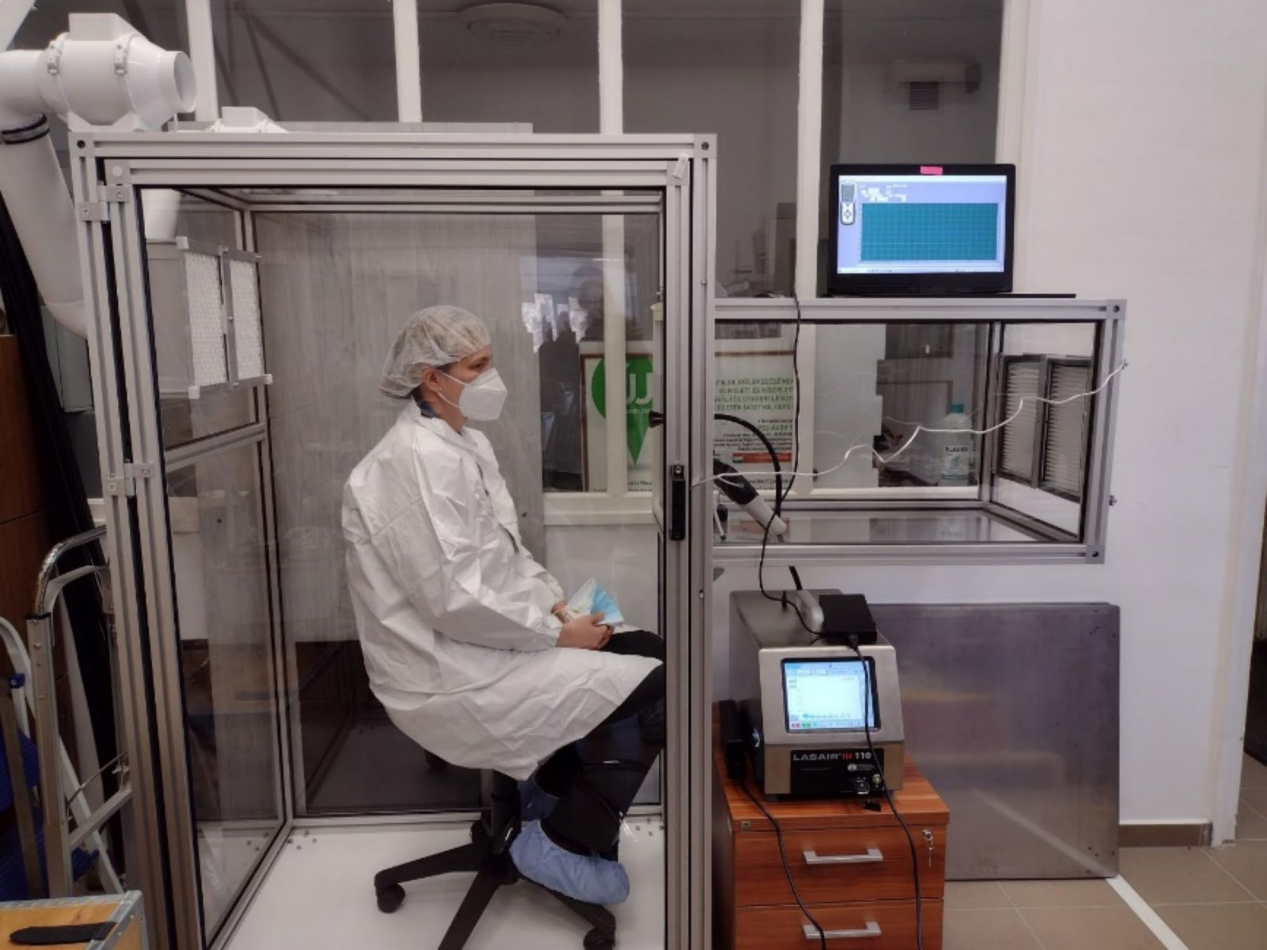
Photograph of the closed measurement cabin and the volunteer during the test.

In order to perform measurements in a clean and controllable environment, which also provides a comfortable seating arrangement for the participating individuals, we designed and constructed a meticulously sealed cabin (Fig. 1). Within this setup, we could arrange the position of the inlet of our measuring device at an optimal location. To ensure the quality of the clean air in the measurement cabin, we have implemented an Ultra-Low Penetration Air (ULPA) filter fan system with ULPA U15 type filters IN18/15 AR (Innofilt Ltd., Budapest). This system operates to establish a clean environment inside the cabin while maintaining a slight overpressure. Our choice of a pressurized system was deliberate, as it effectively hinders the potential influx of additional particles through critical seals. With the help of two Vents TT MIX 100 (Blauberg Group - Vents, Boyarka, Ukraine) industrial pipe fans, generating a maximum volume flow rate of 374 m^3^/h was possible when cleaning the 1.71 m^3^ cabin.

Low-resistance filters were placed opposite the air inlet, through which the air was exhausted. This arrangement created a nearly laminar flow with an average velocity of 0.1 m/s around the subjects’ heads. Before starting the measurements, we mapped the airflow in the cabin using a Voltcraft PL-135 (Conrad Electronic, Hirschau, Germany) hot wire anemometer. The velocity profile was recorded in the perpendicular vertical plane of the inlet of the aerosol measurement instrument in a 7 × 7 grid while the operator sat inside. At each grid point, 25 velocity data were recorded and averaged, and the standard deviation was calculated. The mean velocity was 0.1 m/s around the samplers, and the standard deviation was 0.02. The fact that the velocity does not vary rapidly near the inlet in space and time and the value of the standard deviation is low indicates that a stable, nearly laminar flow field is established in the chamber during the measurements. This 0.1 m/s flow velocity is approximately the same as the velocity of the air sampled by the optical particle counter through the sampling funnel (28.3 lpm flow rate, funnel inlet with 6 cm diameter cross section), ensuring near isokinetic sampling. This sampling arrangement is similar to that described in^11^.

Of course, while the flow field limits the spread of the particle cloud, we cannot determine the total number of particles emitted with this arrangement; only information about particles moving forward can be measured, unlike the full-face masking arrangement used by Bagheri et al.^21^. Our setup provides information about the concentration and size distribution of the particle cluster moving towards the person facing the speaker. In this way, we are characterizing the particle load of the bystanding person rather than aiming at the collection of all the particles. However, this does not affect our scope of investigation of the temporal variation of particle emission and the effectiveness of the masks worn by the individual.

To perform particle size distribution measurements, we used a PMS LasAir III 110-type optical particle counting device (Particle Measuring Systems, Inc., USA). During the experiments, the instrument was configured to produce averages per 6 s of particle number concentrations across eight distinct channels representing particle size intervals of 0.1–0.15, 0.15–0.20, 0.20–0.25, 0.25–0.3, 0.3–0.5, 0.5–1.0, 1.0–5.0 and > 5 μm. The inlet of the LasAir device was positioned at a 20-centimetre horizontal distance from the mouth of the speaker. A Voltcraft SL-451 (Conrad Electronic, Hirschau, Germany) sound level meter was placed adjacent to this setup to monitor sound pressure levels accurately. To monitor the indoor environmental conditions, temperature, humidity and air pressure were recorded once for each volunteer during the measurement.

In order to eliminate the particle emission from the clothing and breathing of the examined person, the individual was dressed in protective clothing, which consisted of a standard medical hairnet, FFP2 mask (Noukang, China), clean cloak and shoe protectors.

The masks used most commonly during the 2019–2021 pandemic period were tested: (i) FFP2/N95 standard mask, (ii) blue surgical mask (Yangyan, China), and (iii) 2-layer cotton mask.

The participants were all healthy adult volunteers without acute respiratory diseases, and none of them were smokers. The group of participants consisted of 13 women and 15 men, spanning an age range from 18 to 56 years old. The average age of men was 33 years (with a standard deviation of 11.5 years), while for women, it was 37 years (with a standard deviation of 11.5 years).

We measured the particle emission and speaking characteristics of 28 adults during standard speech in 4 situations: without any masks or wearing one of the three different mask types. Adhering to our measurement protocol, we instructed the individuals to perform five distinct tasks in the following order:

1. normal speaking without wearing a mask,
2. normal speaking while wearing an FFP2 mask,
3. normal speaking while wearing a surgical mask,
4. normal speaking while wearing a 2-layer textile mask,
5. a control measurement of normal speaking without a mask again.

During speaking, the participants had to count from 1 to 100. Based on the instructions of the surveyor, they had to wait a few minutes between the tasks to achieve the highest degree of clean air in the cabin.

### Ethical approval

This study involved human participants and was approved by the Semmelweis University Ethical Board (ID: 114/2024). Participants gave informed consent to participate in the study before taking part. Informed consent for publication of identifying information was obtained from the participant shown in Fig. 1.

###  Data evaluation

**Fig. 2:**
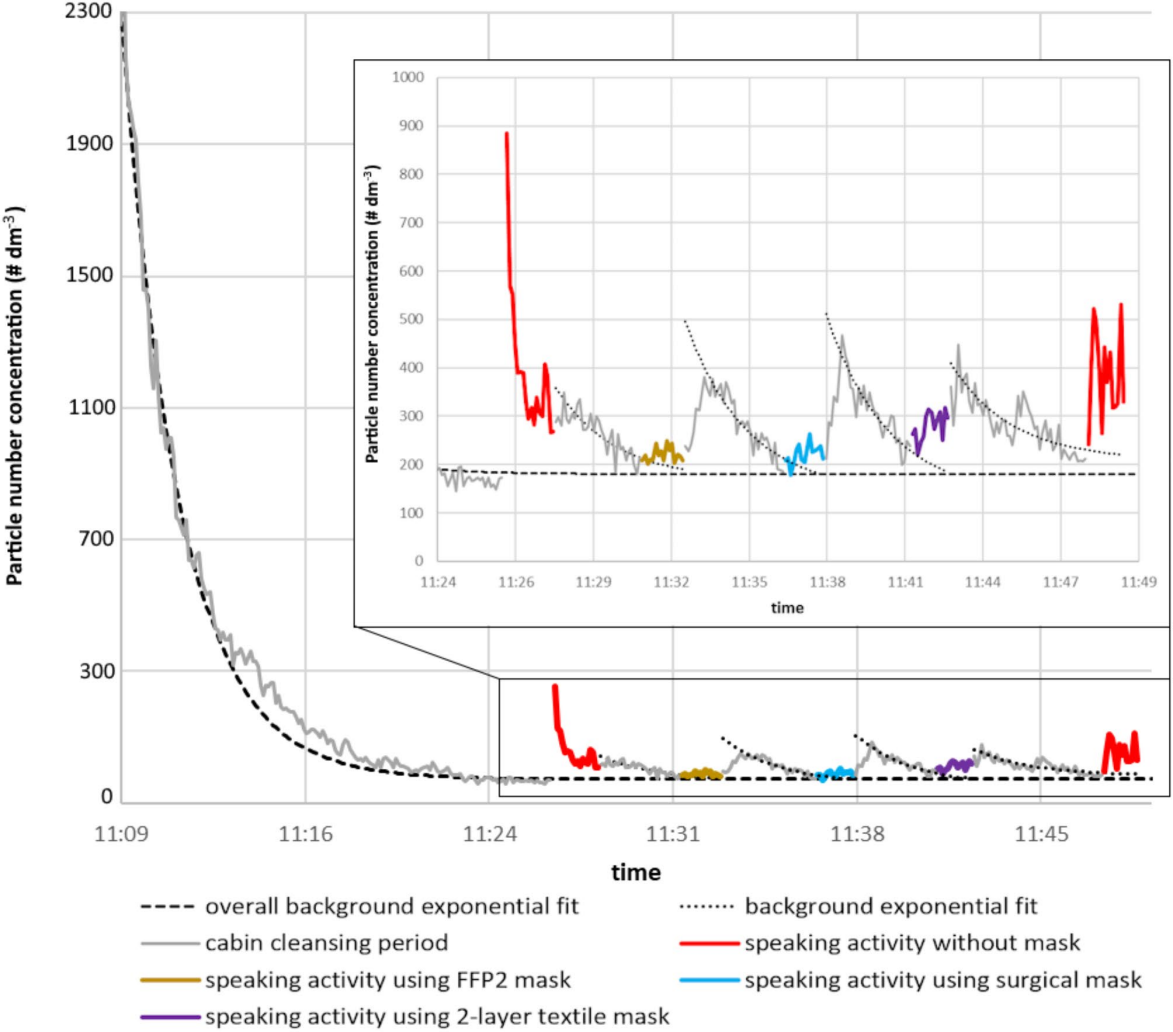
An example of the temporal variation in the measured particle count trend during the full measurement cycle: the volunteer performs the five distinct tasks, with highlighting background concentration. The inset highlights the speaking activities in particular.

Attaining absolute purity without any aerosol contamination inside the cabin was not feasible. Therefore, it was necessary to determine the background concentrations when quantifying the measurement data. Once the individual undergoing the test comfortably postured within the cabin, a 10–15-minute-long cleaning was carried out. Figure 2 illustrates the measured particle count time trend of an entire measurement cycle (five speaking activities and clearance periods between them), highlighting the background correction. The concentration of particles within the cabin exhibited an exponential decline due to the ventilation. In the first step, we fitted a 3-parameter (*A*, *R*, *c*) exponential curve.

to the first longer cleaning period, which we then used to correct for the measurement cycle (subtract it from the measured values). This overall background (*GB*^*ch*^) is marked with a black dashed line in Fig. 2. In the second step, we also had to consider emissions related to the different speaking activities, mask changes, and indoor movements between the performed tasks. Thus, we had to take into account new exponential functions (*B*_*i*_^*ch*^) for each cleaning period preceding each (*i*-th) activity (black dotted lines).

In summary, the corrected concentration (*C*_*i*_^*ch*^) of a certain activity of the *i*-th task for each participant and each *ch* channel is determined using the following formula from the raw measured concentration value (*Raw*_*i*_^*ch*^):

$$\:{C}_{i}^{ch}\left(t\right)={Raw}_{i}^{ch}\left(t\right)-{GB}^{ch}\left(t\right)-{B}_{i}^{ch}\left(t\right)$$

Since the timing of the start of the speaking activities was set so that the background concentration approached the minimum based on our experience, we can assume that the speech occurred during the phase of the exponential decay of the background concentration, where it is already approximately linear:

$$\:{GB}^{ch}\left(t\right)=\frac{f\left({k}_{5}\right)-f\left({w}_{1}\right)}{{k}_{5}-{w}_{1}}\left(t-{w}_{1}\right)+f\left({w}_{1}\right)$$

and

$$\:{B}_{i}^{ch}\left(t\right)=\frac{f\left({k}_{i}\right)-f\left({w}_{i}\right)}{{k}_{i}-{w}_{i}}\left(t-{w}_{i}\right)+f\left({w}_{i}\right)$$,

where *w*_*i*_ and *k*_*i*_ are the starting and ending point of the *i*-th activity.

##  Results and discussion

It is already known that particles emitted from the human lungs undergo a significant size decrease as they evaporate upon entering the atmosphere, a phenomenon strongly influenced by environmental conditions^26^. While we did not actively aim to keep the environmental conditions constant throughout the measurements, we observed minimal variation in the atmospheric parameters within the cabin during the measurement series. The measurements conducted inside the cabin revealed a relative humidity ranging from 34 to 39%, along with a temperature detected between 21 °C and 24 °C. In the work of Xie et al.^27^, a simple physical model is introduced to investigate the evaporation and movement of droplets expelled during respiratory activities. Their findings show that particles smaller than 10 μm dry out within 1 s in the case of a normal indoor environment (20 °C < *T* < 33 °C and 30% < *RH <* 70%). As the particles travel much longer than this time period between the inlet and the mouth and through the 1-meter-long intake tube, we can assume during the evaluation of the measurement data that the tested submicron-sized particles reached their equilibrium size.

### Number size distribution of particles emitted during speaking

**Fig. 3:**
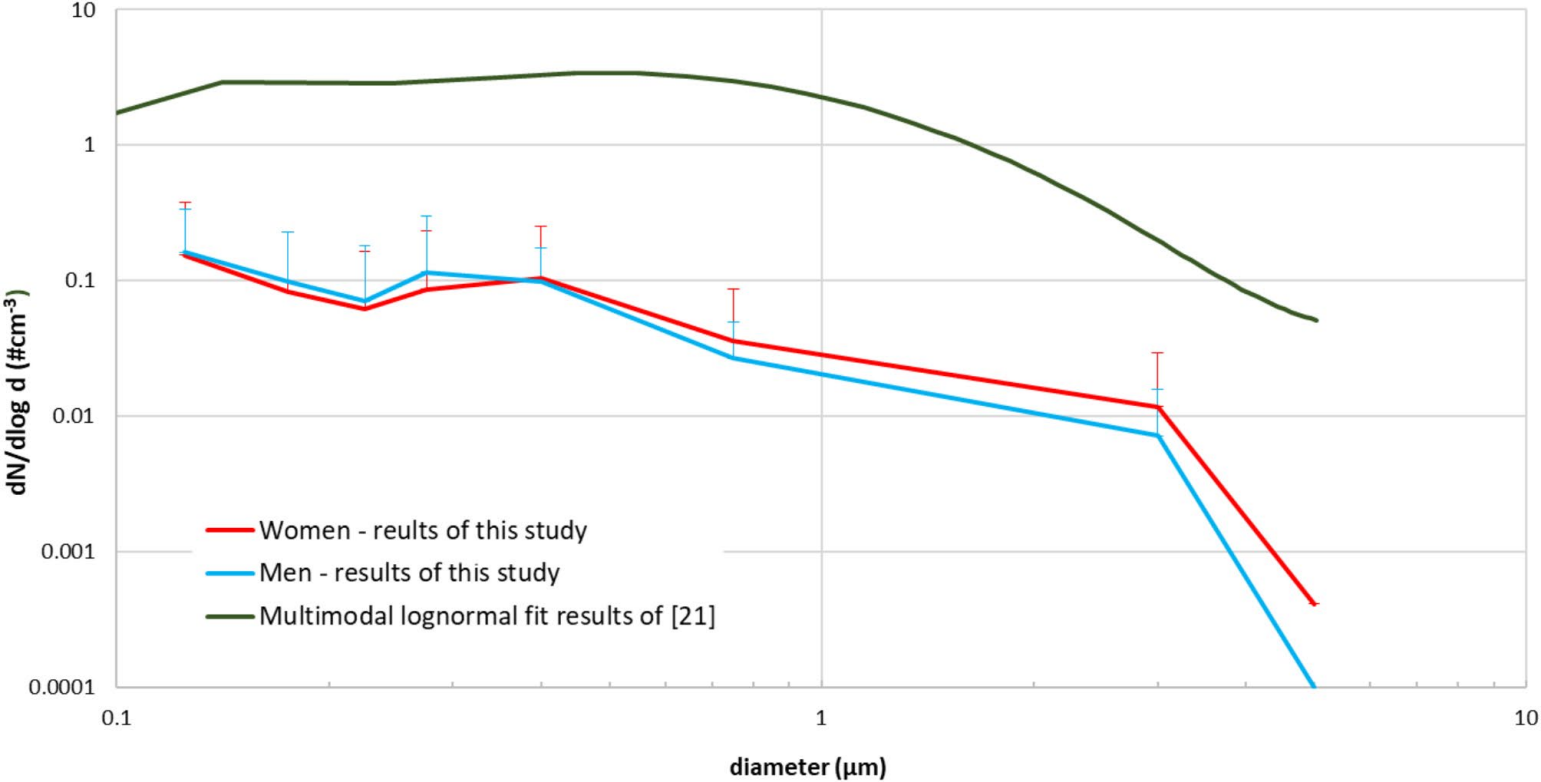
Measured average size distribution of particles emitted by speaking individuals showing intersex differences. Data from the open literature are also shown for comparison. The error bars indicate the standard deviation values.

The number size distributions of exhaled particles were examined twice during the speaking activity of each of the 28 volunteers, as the measurement protocol intended the repetition of counting at the beginning and the end. Figure 3 demonstrates the variability among participants, showing the total average number size distributions and the standard deviation of the measured values.

It is evident that the number size distribution of emitted particles in the submicron range exhibits a multimodal pattern, reflecting contributions from distinct respiratory regions and mechanisms. Research results summarized in^18 and 23^ indicate that a generalized parametrization based on three log-normal modes accurately represents the experimental data within the submicron range. Two of these modes (approx. 0.1 μm and 0.3 μm modes), originate from the bronchiolar region, primarily associated with the BFFB mechanism, where cyclic airway closure and reopening lead to the rupture of epithelial lining fluid films deep within the lungs. Such particles are predominantly formed in the alveolar and small bronchiolar airways and are consistently present during normal respiration.

Beyond these bronchiolar contributions, a third mode (LT), ranging in a wide range from 0.5 to 1.5 μm, emerges, likely originating from the larynx and trachea. This mode is linked to laryngeal particle generation, where vocal fold adduction and vibration induce high-shear stress at the ELF-air interface, leading to particle detachment. This mechanism is particularly active during vocalization, coughing, and sneezing, where turbulent airflow enhances particle formation.

The detected particle number size distribution (see Fig. 3) provides significantly more pronounced results compared to measurement data available in the literature^18,21,23^), refining the distribution in the submicron range and highlighting the dominance of particles in the 100–150 nm size range. Additionally, a secondary peak appears (300 nm mode) in the fine structure, suggesting that the size distribution of emitted particles and the mechanisms determining it can be more precisely characterized during speech. Such particles are predominantly formed in the alveolar and small bronchiolar airways and are consistently present during normal respiration, as well as during speaking and singing, as breathing is inherently involved in these activities, which even emphasizes the importance of BFFB mechanism during speaking.

The sample size available allowed us to study intersex differences. In Fig. 3, the average values for men and women are shown by blue and red lines, respectively. It can be unequivocally affirmed that sex, specifically the height of a tone, does not influence either the size distribution or the quantity of emitted particles significantly. However, it is noteworthy that there is substantial individual variability in particle quantity, with variations of up to two orders of magnitude observed at comparable sound levels.

### Changes in the number of emitted particles over time during speaking

Apart from assessing the size distribution of emitted particles, it is crucial to understand and quantify how this distribution of submicron particles evolve over time. Our measurement setup, ensuring high time resolution (6 s) and sensitivity, allowed us to explore the temporal dynamics of particles during speech. We studied the temporal trends of emitted particles over 60–120 s for all participants while counting from 1 to 100.

**Fig. 4:**
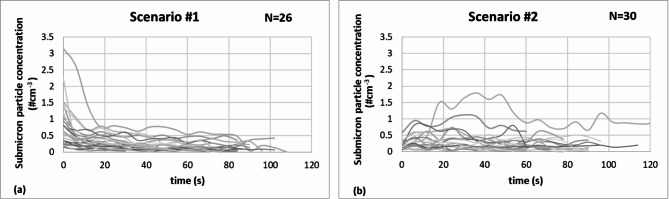
Time trend of the number concentration of the total emitted submicron particles during continuous speaking without wearing a mask, where panels (a) and (b) show the curves corresponding to the two different scenarios. We indicated the number of curves (N) in the figure in the top right corner classified to each scenario.

The measurement results show interesting differences among individuals and suggest a general trend of decreasing submicron particle number concentration during continuous speech. Based on the short (< 120 s) measurements, it was found that the time trends of individual participants could be categorized into two distinct groups, called scenarios [Fig. 4 (a) and (b)]. In the first scenario [Figure 4 (a)], the number concentration of particles emitted during speaking is clearly characterized by a decreasing trend. In nearly half of the cases, a substantial particle emission occurs at the speech’s onset, followed by a gradual decline. In the second scenario [Figure 4 (b)], the temporal pattern diverges from the preceding ones, with a subtle decrease in the number of particles following an initial increase or displaying a lack of temporal trend and experiencing time-independent emissions. For nine cases of speaking without wearing a mask, the time trend could not be specified since the concentration values related to emission could not be distinguished from the background concentration values at several measurement points within the speaking period.

Conducting the measurement twice for each volunteer allows for the exploration of individual variability. Based on the above-defined scenarios, a consistent trend was found for 61% of the volunteers, while the remaining participants exhibited variations in their emission patterns.

A fundamental prerequisite for risk assessment is a precise and comprehensive database, which provides the basis for conducting a robust health impact assessment^28–31^. The variability between super-emitters and low particle emitters significantly modifies the probability of the potential impact by creating pollution hotspots, leading to underestimation of localized risks when relying on average emissions. Super-emitters contribute disproportionately to overall pollution, causing spatial and temporal exposure variability, which challenges traditional risk modeling. A qualitative analysis of the effect of “super emissions” on the associated infection risk could be done by the use of available risk models, such as the online ‘Virus Tool’, presented in Riediker and Monn^32^. Our results indicate that human groups with similar emission characteristics can be distinguished, providing an opportunity for further risk assessment refinement.

Moreover, when considering individual variability, it is valuable to take into account the temporal aspect of particle emission; for example, a notable trend of emitting a high number of particles at the outset, followed by a substantial decrease in subsequent values in the case of speaking activities belonging to scenario #1. As highlighted by Morwaska et al.^4^, a much better comprehension of the dynamics during the initial phases of the respiratory plume is essential. Thus, the first few seconds play a critical role in the airborne transmission of respiratory pathogens, especially in close proximity situations.

It is also important to know whether there is a change in the size distribution of the emitted particles during speaking activity. To study this, we statistically evaluated the bimodal distribution of the instantaneous size distributions, examining in which size range the first and second maxima were located. Based on this, considering all (*N* = 781) curves corresponding to speech without a mask at all measurement points during the speaking activity, it can be determined that the first maximum fell within the 100–150 nm size range in 98% of the samples, while the second peak was located in the 300–500 nm range in 90.5% of the cases. The remaining less than 10% is distributed approximately equally between the adjacent size ranges. Thus, based on our measurements, we can conclude that no changes were detected in the submicron range, and the bimodal size distribution remains characteristic until the end of the speech in both scenarios.

**Fig. 5:**
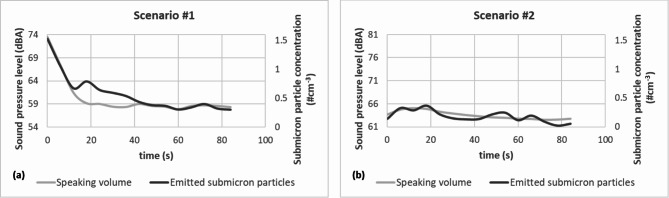
Time trend of the number concentration of total emitted submicron particles and speaking volume during continuous speaking. Panels (a) and (b) show the curves corresponding to the two scenarios.

Sound pressure levels (dBA) were registered with a 1-second resolution throughout the measurements. All participants were instructed to speak at their standard volume. In order to study the dependence of the number of emitted particles on speaking volume, the sound pressure levels were averaged over 6-second-long periods. An example for each above-defined scenario is shown in Fig. 5.

It can be observed that there is a consistent trend of decreasing volume during speaking, coinciding with a reduction in the particle count. Simultaneously, the time trends of submicron particle emissions are similar to sound pressure level changes in general. However, it is important to note that a common relationship cannot be conclusively demonstrated.

Therefore, it can be stated that the temporal duration of the measurements holds a significant role in our investigations when investigating the determining effects of the quantity of emitted bioaerosols.

However, one has to consider various factors as the turbulent aerosolization process depends on many parameters (such as liquid surface tension, air density, airstream velocity, the viscosity of the media, etc.), which shows beyond the present research, nevertheless, highlights the importance of considering the temporality.

### Source control efficiency of masks in the submicron size mode

During the recent pandemic, the use of diverse masks emerged as an effective supplementary defence strategy in mitigating human particle emissions^33–35^. Source control efficiency is defined as the percentage of aerosol particles blocked by the face mask compared to experiments conducted without a mask^36^. It can be assumed that this percentage represents the fraction of particles removed from the air passing through the mask material. While a load of measurement data exists on the filtering efficiency of various masks, there is a scarcity of in vivo tests aligning with real-world wearing habits. Consequently, our research delved into studying the release of aerosol particles into the environment while wearing the most prevalent masks in the European context.

**Fig. 6:**
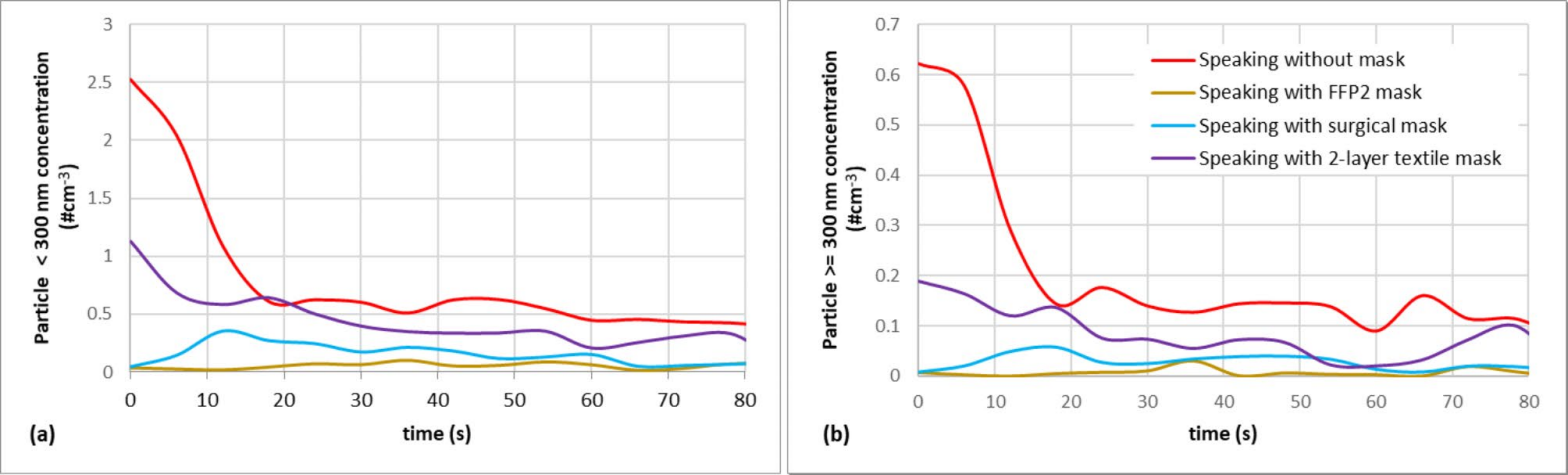
Time trends of particle concentration in the case of a selected volunteer for (a) particles under and (b) above or equal to 300 nm for all speaking tasks.

In our measurement protocol, we studied the volunteers’ emission while speaking and evaluated the source control efficiency when wearing three different masks, as detailed earlier in “Measurement setup”. Wearing habits influence the performance of the masks, so our measurement results represent the source control efficiency under real-life conditions rather than filtering efficiency in regular sense. As the speech of the volunteers in the various tasks cannot be considered completely identical, we need to assess first whether different source control efficiencies can be observed in different particle size ranges and whether the number of emitted particles or the speech properties (speaking volume) have a larger influence on the mask’s source control efficiency. Figure 6 presents the temporal variation of particle concentration detected during the speech of one volunteer with and without masks. Our results show, in general, that although the speeches are not identical when wearing different masks, the capture of particles is effective in real-life conditions for all types of masks and even in the case of initially high emitted particle concentrations, the number of particles entering the atmosphere remains appropriately low.

Additionally, our results show that the filtration efficiency is the most stable in the case of the FFP2 mask, capturing the initially high emitted concentration. In contrast, a more gradual reduction over time is observed for textile masks.

For quantifying source control efficiency under real conditions, we chose a statistical evaluation, keeping in mind that each speech is different and shows a varying trend over time. For this, we determined the ratio of the number of particles in each size fraction based on the measured concentration while speaking without a mask and with a mask. For the *i*-th voluntary, for each size channel (*ch*), the instantaneous mask source control efficiency (*Eff*^*ch*^) was determined as follows based on the measured number concentration in case of wearing a mask (*Mask*^*ch*^) and without wearing a mask (*Raw*^*ch*^):

$$\:{Eff}_{i}^{ch}=\frac{{Mask}_{i}^{ch}(\varDelta\:{t}^{*})}{{Raw}_{i}^{ch}(\varDelta\:t)}$$

where *t*_0_^*^ and *t*_*0*_ are the starting moment of activities with and without wearing a mask, respectively, but Δt^*^ = *Δt*.

This was then averaged for all participants (*p =* 28) and through the entire speaking activity for each size channel. It is important to note that the durations of different activities were not always the same. Therefore, we always took the shorter one. That is, if we had *n*^∗^ and *n* measurement points in time, we averaged over =min{*n*^∗^;*n*} points:

$$\:mask\:efficiency=\sum\:_{n,\:i}\frac{{Eff}_{i}^{ch}}{l*p}$$

The source control efficiency corresponding to each mask in the examined size ranges is presented in Table 1. Our findings revealed that FFP2 and surgical masks demonstrated a great performance across the entire submicron size spectrum, including particles below 300 nm (72–87%, rel. st. dev. 26–60%). Literature findings regarding FFP2 masks reveal a source control efficiency exceeding 98% within the size range of 10–700 nm^23,25,37–39^, whilst, in the case of surgical masks, a very close, but slightly lower source control efficiency was found.

**Table 1:** Source control efficiency results under real-world wearing conditions based on in vivo experiments. Reference data are all from controlled laboratory experiments.

Size (nm)	FFP2 mask		Surgical mask		2-layer textile mask	
	Average source control efficiency (%) and std. deviation	Reference filtering efficiency (%)	Average source control efficiency (%) and std. deviation	Reference filtering efficiency (%)	Average source control efficiency (%) and deviation	Reference filtering efficiency (%)
100	0.82 ± 0.24	0.8-1^39^ 0.99^38^ 0.98^25^	0.72 ± 0.60	0.98^38^ 0.6–0.8^39^ 0.82^40^ 0.3^25^	0.56 ± 0.58	0.08^25^
150	0.87 ± 0.20		0.85 ± 0.23		0.63 ± 0.49	
200	0.77 ± 0.26		0.82 ± 0.31		0.59 ± 1.00	
250	0.76 ± 0.35	0.98^25^	0.75 ± 0.31	0.22^25^ >0.99^23^	0.49 ± 0.83	0-0.2^23^ 0.38^25^
300	0.80 ± 0.35		0.83 ± 0.30		0.68 ± 0.43	
500	0.74 ± 0.40		0.78 ± 0.53		0.48 ± 0.67	
1000	0.87 ± 0.55	> 0.99^23^	0.88 ± 0.56	> 0.99^23^	0.46 ± 0.69	0.3^23^
5000	0.47 ± 1.00	0.99^23^	0.38 ± 1.13	0.99^23^	0.40 ± 0.80	0.5 ^23^

As shown in Table 1, higher source control efficiency was observed in experiments conducted under controlled conditions for FFP2 compared to those conducted under real-world wearing conditions. This difference is clearly attributable to leakage caused by improper fit to the face. At the same time, it should be noted that the results are subject to errors inherent in the measurement process, as no two speech-related emission time profiles are identical, as shown in “Changes in the number of emitted particles over time during speaking”.

In the case of surgical masks, the literature data vary within a very wide range. Some studies show high^23,38,40^, whilst results shown in^25^ report significantly worse source control efficiency, discussing the benefits of the layered mask structure. Based on our results, it can be concluded that the source control efficiency of a surgical mask in the submicron size range is similar to that of an FFP2 mask under real-world wearing conditions but only around 80%.

The source control efficiency of 2-layer textile masks is lower, 49–56% in the particle size range under 300 nm, and even worse, 40–48% for particles larger than 300 nm. According to LaRue et al.^37^, there is no discernible distinction between real and non-real experimental setups for these masks.

It is important to highlight that these masks exhibited their poorest performance within the 1–5 μm size range. This can be attributed to the inadequacy of sample size within this specific range, necessitating prolonged sampling for more accurate refinement.

The considerable variance in measurement outcomes distinctly underscores the impact of individual mask-wearing habits, such as the fitting precision. It illuminates how diverse and demonstrable these habits influence the quantity of particles released into the environment.

## Conclusion

In this study, our focus was on the characterization of respiratory particles within the submicron range (100–1000 nm), given the limited availability of comprehensive literature results. Throughout the in vivo experiments presented, we not only examined the number size distribution of emitted particles but also delved into the temporal evolution of particle emissions from the human body during continuous speech as volumetric particle emission rate is an essential component of the modelling of airborne transmission risk assessment.

Our findings indicate that the size distribution of submicron particles originating from the lungs and upper airways exhibits a consistent bimodal pattern, with modes around 300 nm and below 100 nm, which is an addition to the sparse results summarised in the work of Bagheri et al.^23^. While the volume of these particles typically undergoes significant changes during continuous speech, the magnitude of this variation is individual-specific. Notably, in 22% of cases, super-emitters (individuals who release a substantial quantity of particles during speech) were identified with a subsequent significant reduction in emission as speaking continues. Since an essential requirement for risk assessment is the precise knowledge of input values and their variability, mapping individuals with different emission profiles can significantly improve the accuracy of health impact assessments, including infection spread and risk analysis. Thanks to the high temporal resolution of our investigation, we have demonstrated that particle emissions during speaking activity could exhibit significant temporal variation (typically a decrease in time), which further refines the evaluation of the impact of super emitters.

While a general decline up to a certain value in the total particle count is characteristic, the tendency is not always sort of linear during prolonged speaking activity. Occasionally, local increases in particle emission were observed towards the latter part of speeches. Furthermore, our study showcased a remarkable sensitivity to speech volume, underscoring the crucial role of high-frequency measurements in comprehensively investigating various factors influencing respiratory particle emissions. Although the full comprehension of this phenomenon extends beyond the scope of our investigations, it is crucial to understand it so that more precise modelling of risk assessments could be achieved, particularly given the inherent temporal dynamics of bioaerosol emissions in real-life situations.

A new and medically important finding is that surgical masks significantly decrease the emitted submicron particles during normal speech. Although the COVID-19 pandemic has led to numerous experimental setups examining the filtration efficiency of masks, and some of them are marketed based on standards, there is still no available data on their filtration efficiency in real in vivo conditions simulating medical ward consultations or longer surgeries concerning the size distribution of emitted particles. Additionally, the results from controlled laboratory setups vary within a wide range, especially in the case of blue surgical masks, highlighting the importance of measurements conducted under in vivo conditions. Our data are reassuring that wearing a surgical medical mask decreases the number of submicron particles emitted, which - when carrying pathogens - reduce the risk of surrounding individuals from airborne infection. Results also show that the FFP2 and surgical masks consistently reduce the number of emitted particles to a very low level, even in cases of exceptionally high short-term emissions, across all examined submicron size ranges.

Our study also has limitations, as the long-term use of the mask was not tested, and further studies are needed to gather information about changes in speech-emitted particles following the longer wearing of a given mask. The characterization of different particle emitters with varying health impacts requires a larger series of measurements.

## Data Availability

The data related to the present study can be obtained from the corresponding author A. Nagy (nagy.attila@wigner.hun-ren.hu), upon reasonable request.
